# Mobile Health Technology Use and the Acceptability of an mHealth Platform for HIV Prevention Among Men Who Have Sex With Men in Malaysia: Cross-sectional Respondent-Driven Sampling Survey

**DOI:** 10.2196/36917

**Published:** 2022-07-25

**Authors:** Roman Shrestha, Francesca Maviglia, Frederick L Altice, Elizabeth DiDomizio, Antoine Khati, Colleen Mistler, Iskandar Azwa, Adeeba Kamarulzaman, Mohd Akbar Ab Halim, Jeffrey A Wickersham

**Affiliations:** 1 Department of Allied Health Sciences University of Connecticut Storrs, CT United States; 2 Section of Infectious Diseases Yale University New Haven, CT United States; 3 Faculty of Medicine University of Malaya Kuala Lumpur Malaysia

**Keywords:** HIV, mHealth, men who have sex with men, mobile phone, Malaysia, mobile health, HIV prevention, sexual health, public health, digital health, communication technology, health technology, technology accessibility, smartphone app, HIV treatment

## Abstract

**Background:**

The growth in mobile technology access, utilization, and services holds great promise in facilitating HIV prevention efforts through mobile health (mHealth) interventions in Malaysia. Despite these promising trends, there is a dearth of evidence on the use of mHealth platforms that addresses HIV prevention among Malaysian men who have sex with men.

**Objective:**

The goal of this study was to gain insight into (1) access and utilization of communication technology (eg, landline phone, internet, mobile phone), (2) acceptability of mHealth-based interventions for HIV prevention services, and (3) preferences regarding the format and frequency of mHealth interventions among Malaysian men who have sex with men.

**Methods:**

We conducted a cross-sectional survey with Malaysian men who have sex with men between July 2018 and March 2020. Participants were recruited using respondent-driven sampling in the Greater Kuala Lumpur region of Malaysia. We collected information on demographic characteristics, HIV risk-related behaviors, access to and the frequency of use of communication technology, and acceptability of using mHealth for HIV prevention using a self-administered questionnaire with a 5-point scale (1, never; 2, rarely; 3, sometimes; 4, often; 5, all the time).

**Results:**

A total of 376 men participated in the survey. Almost all respondents owned or had access to a smartphone with internet access (368/376, 97.9%) and accessed the internet daily (373/376, 99.2%), mainly on a smartphone (334/376, 88.8%). Participants on average used smartphones primarily for social networking (mean 4.5, SD 0.8), followed by sending or receiving emails (mean 4.0, SD 1.0), and searching for health-related information (mean 3.5, SD 0.9). There was high acceptance of the use of mHealth for HIV prevention (mean 4.1, SD 1.5), including for receiving HIV prevention information (345/376, 91.8%), receiving medication reminders (336/376, 89.4%), screening and monitoring sexual activity (306/376, 81.4%) or illicit drug use (281/376, 74.7%), and monitoring drug cravings (280/376, 74.5%). Participants overwhelmingly preferred a smartphone app over other modalities (eg, text, phone call, email) for engaging in mHealth HIV prevention tools. Preference for app notifications ranged from 186/336 (53.9%), for receiving HIV prevention information, to 212/336 (69.3%), for screening and monitoring sexual activity. Acceptance of mHealth was higher for those who were university graduates (*P*=.003), living in a relationship with a partner (*P*=.04), engaged in sexualized drug use (*P*=.01), and engaged in receptive anal sex (*P*=.006).

**Conclusions:**

Findings from this study provide support for developing and deploying mHealth strategies for HIV prevention using a smartphone app in men who have sex with men—a key population with suboptimal engagement in HIV prevention and treatment.

## Introduction

In comparison with high-income countries, low- and middle-income countries have disproportionately high rates of HIV because funding and access to HIV prevention and treatment services are limited [1]. Men who have sex with men are at increased vulnerability to HIV due to a combination of biological factors (ie, sexual exposure risk, concurrent sexually transmitted infections) [2] and a higher sexual network or community HIV prevalence [3,4]. In addition, behavioral factors (eg, condomless sex, multiple sexual partners, sexualized drug use which is also known as *Chemsex*) have also been found to increase the risk of HIV in this group [4,5].

In Malaysia, HIV prevalence among men who have sex with men has grown exponentially in the past decade, peaking at 21.6% in 2017, compared with 0.4% among the general population [6,7]. The causes of Malaysia’s expanding HIV epidemic among men who have sex with men are multifactorial. In populations such as men who have sex with men, the uptake of HIV prevention and treatment services is often low due to reluctance to disclose their sexual orientation, lack of anonymity, and concerns about confidentiality [8]. In Malaysia, which is a middle-income country with a Muslim majority, same-sex sexual behavior is a crime according to both secular and Sharia criminal laws, which contributes to high levels of stigma and discrimination. The criminalization of same-sex relationships has been found to be associated with lower access to condoms, lubricants, HIV testing, and HIV treatment in a study [9] with a sample of men who have sex with men from 115 countries. Several studies [10-13] have documented negative attitudes and discrimination toward men who have sex with men in Malaysia, including in health care settings. Consequently, the scale-up of evidence-based HIV testing, prevention, and treatment programs that target those who are most vulnerable to HIV, including men who have sex with men, has been negatively impacted. Low HIV-testing uptake among men who have sex with men in Malaysia has resulted in many men who have sex with men being diagnosed with HIV at an advanced stage [8,14-16]. There is, therefore, a need for innovative strategies to reach with services and information and retain into care stigmatized populations such as men who have sex with men in Malaysia.

The use of mobile health (mHealth) technology to improve health outcomes has been expanding globally, and research is needed to guide its use in health care delivery [17-23]. mHealth has been found to be a promising and cost-effective strategy to reach and serve stigmatized and hidden populations such as men who have sex with men [24-27]. In Malaysia, leveraging an mHealth platform may allow users to feel safer and less vulnerable to potential legal or social harm (for example, by reducing face-to-face interactions with providers) and bypass barriers to care for marginalized populations [28-32]. Over the past decade, the use of mobile technology in Malaysia has grown markedly, with a mobile phone penetration rate of 97.5% and an internet penetration rate of 71.1% [33]. With increases in access to, utilization of, and services using communication technologies, an mHealth approach holds great promise to facilitate HIV prevention efforts in Malaysia.

Despite these promising trends, there is a dearth of evidence on the use of mHealth platforms to address HIV prevention in Malaysian men who have sex with men. Thus, the goal of this study was to gain insight into (1) access to and utilization of communication technology (eg, landline phone, internet, cell phone), (2) acceptability of mHealth-based interventions for HIV prevention services, and (3) preferences regarding the format and frequency of mHealth interventions among Malaysian men who have sex with men.

## Methods

### Study Design and Setting

We conducted a cross-sectional study with participants recruited from the Greater Kuala Lumpur region between July 2018 and March 2020. Data collection was conducted in a private room at the Centre of Excellence for Research in AIDS (University of Malaya), because of its central location in the Greater Kuala Lumpur region, accessibility by public transport, and ability to provide free parking to participants.

### Study Participants and Procedures

Inclusion criteria were (1) being 18 years or older; (2) identifying as a cisgender male; (3) currently residing in the Greater Kuala Lumpur region; (4) reporting having been engaged in sexual activity with male sexual partners in the previous 6 months; and (5) being able to read and understand English, Chinese, or Bahasa Malaysia.

The questionnaires were created in English and then translated to Chinese and Bahasa Malaysia. We used forward-backward translation and pilot tested the translated questionnaires to ensure translation quality.

Participants were recruited using respondent-driven sampling, which is a network-based sampling method that is often used for hard-to-reach populations and combines peer-driven recruitment and statistical adjustments to reduce bias and approximate random sampling [34]. During our formative work, initial respondent-driven sampling participants, called seed participants, were carefully selected with assistance from community-based organizations for men who have sex with men. We aimed to recruit a seed participant sample (n=27) that reflected the diversity (eg, ethnicity, age) of the community of men who have sex with men in the region.

Each participant who completed the study was given 3 coupons to recruit men from their peer networks, who were, in turn, also given 3 coupons. Each coupon card contained a unique respondent-driven sampling number that allowed us to trace the peer recruitment chain and important study-related information (study site address, contact information for the study team, and inclusion criteria). Coupon management software was used to track distributed and redeemed coupons during the study, and a standard numbering system was used to track the recruiter-recruit relationship. Participants received 30 Malaysian Ringgit (at the time of publication, 1 MYR was approximately equivalent to US $0.23) for study participation and an additional 10 MYR for each peer who was successfully recruited to the study (up to 3 peers).

Individuals could choose whether to come during walk-in hours or set an appointment by phone. The research site was open 7 days a week to offer maximum flexibility and accommodate different work schedules. Individuals who presented with a valid coupon underwent initial eligibility screening. If eligible, they were asked to provide informed consent. Each participant completed the web-based questionnaire (Qualtrics, Qualtrics XM) in a private room, which took approximately 20 minutes, while study staff waited outside the room, to ensure privacy.

### Ethics Approval

The study was approved by the institutional review boards of the University of Malaya (201854) and Yale University (2000023152).

### Measures

#### Participant Characteristics

We collected participant characteristics (age, ethnicity, educational status, relationship status, income, history of childhood physical abuse, history of childhood sexual assault, and depressive symptoms experienced in the past week).

#### Access to and Frequency of Use of Communication Technology

We adapted a scale that we used in previous studies [35-37] to measure participants’ access to and frequency of use of various types of communication devices, including landline telephone, mobile phone with internet access (smartphone) and without internet access (basic phone), tablet, laptop, and personal computer. Participants were asked how often they use each technology on a 5-point Likert scale (ranging from 1, never, to 5, all the time).

Additionally, participants were asked if they had daily access to the internet, which device was their primary device for accessing the internet, and the number of hours spent on the internet each week. Participants’ utilization of smartphones for various internet-based activities (including social networking, sending or receiving emails, using geosocial networking apps or websites, searching for health-related information, or using health-related apps) was assessed using a 5-point Likert scale (ranging from 1, never, to 5, all the time). Participants were also asked which men who have sex with men–related geosocial networking apps or websites (eg, Grindr, Hornet, Planet Romeo) they currently used.

#### Acceptability of mHealth

The mHealth acceptance scale was adapted from previous studies [35-37]. Participants were asked about their willingness to use 5 mHealth-related features—receiving medication reminders, monitoring drug cravings, screening and monitoring illicit drug use, screening and monitoring sexual activity, and receiving information about HIV prevention. Each feature was rated on a 5-point Likert scale (ranging from 1, not willing, to 5, extremely willing); the scale was later dichotomized for analysis, with “not willing” coded as *no* and “somewhat willing,” “willing,” “very willing,” “extremely willing” coded as *yes*. An mHealth acceptance score was created by taking an average cumulative score of the 5 mHealth-related dichotomized variables, with a higher score indicating greater acceptance (α=.88). Respondents’ preferred frequency (ie, daily, weekly, and monthly) and modality of mHealth (ie, phone call, text message, email, or app) were also assessed [35-37].

#### Childhood Trauma and Mental Health

Two items from the US Centers for Diseases Control and Prevention’s Behavioral Risk Factor Surveillance System questionnaire [38] were used to measure history of childhood and physical and sexual trauma. Childhood physical trauma was measured with a single-item question, “Before the age of 18, were you ever hit, slapped, kicked, or physically hurt by an adult?” Childhood sexual trauma was measured with 2 items: “Before the age of 18, were you ever forced to have sex by an adult or older child?” and “Before the age of 18, were you ever touched in a sexual way by an adult or older child when you did not want to be touched that way or were you ever forced to touch an adult or older child in a sexual way?” A “yes” response to either question resulted in a *yes* coding for the presence of childhood sexual trauma. Depressive symptoms were assessed using the 10-item Center for Epidemiological Studies Depression scale [39,40]. The total sum score ranges from 0 to 30, with a standard cut-off (score >10) for moderate to severe depression (α=.89) [40].

#### Sexual and Drug-Related Behaviors

Participants were asked information about their sexual behavior, including recent (past 6 months) engagement in anal sex and in which role (ie, insertive or receptive; participants were able to select both roles if applicable); recent engagement in an HIV-serodiscordant sexual relationship; consistent condom use; and lifetime engagement in sexualized drug use, which we defined as any use of crystal methamphetamine, gamma-hydroxybutyrate, gamma-butyrolactone, or 5-methoxy-N, N-diisopropyl tryptamine (commonly known as foxy or foxy methoxy [41]) before or during sexual activity. Additionally, participants were asked about any lifetime injection drug use. The 6-month cut-off point for sexual activity and engagement in a serodiscordant relationship was chosen based on the Centers for Disease Control and Prevention guidelines [42].

Participants were asked if they had ever been tested for or diagnosed with HIV or other sexually transmitted infections, including chlamydia (*Chlamydia trachomatis*), gonorrhea (*Neisseria gonorrhoeae*), syphilis (*Treponema pallidum*), hepatitis B (*Orthohepadnavirus hepatitis B virus*), and hepatitis C (*Hepacivirus hepacivirus C*), and if they had ever used pre-exposure prophylaxis or postexposure prophylaxis for HIV prevention.

### Data Analysis

Analyses were performed using SPSS software (version 26; IBM Corp). We calculated descriptive statistics, such as frequencies and percentages for categorical variables and means and standard deviations for continuous variables, and used multivariate linear regression to assess factors associated with the primary outcome—willingness to use mHealth (measured by the mHealth acceptability scale (continuous variable). Candidate covariates were selected based on previous literature on mHealth acceptability and were included in the multivariable model if *P*<.05 in a bivariate model. Estimates were evaluated for statistical significance based on probability criteria of *P*<.05.

## Results

### Participant Characteristics

A total of 376 men (age: mean 27.5 years, SD 6.5 years) participated in the survey. Most participants identified ethnically as Malay (220/376, 58.5%). Over half of the participants were university graduates (216/376, 57.4%) and single (216/376, 56.4%). The mean monthly income was 3602.9 MYR. The majority of participants (222/376, 59.0%) reported symptoms consistent with moderate to severe depression (Table 1).

Although almost all participants had engaged in anal sex in the past 6 months (363/376, 96.5%; receptive role: 285/376, 75.8%), only one-fifth (72/376, 19.1%) reported consistent condom use. Moreover, one-fifth of participants (82/376, 21.8%) reported having ever engaged in sexualized drug use. Overall, 71.0% (267/376) of the participants had been tested for HIV at least once in their lifetime, and 27.4% (103/376) had been previously diagnosed with a sexually transmitted infection other than HIV. Only a small proportion of participants had ever used pre-exposure prophylaxis (26/376, 6.9%) or postexposure prophylaxis (27/376, 7.2%).

**Table 1:** Characteristics of participants.

Characteristic			Respondents (n=376)
Age (years), mean (SD)			27.5 (6.5)
**Ethnicity (Malaya), n (%)**			
	No	156 (41.5)	
	Yes	220 (58.5)	
**University graduate^a^, n (%)**			
	No	160 (42.6)	
	Yes	216 (57.4)	
**Relationship status, n (%)**			
	Single	212 (56.4)	
	Partner	164 (43.6)	
Monthly income (MYR)^b^, mean (SD)			3602.9 (5082.6)
**Ever had HIV test, n (%)**			
	No	109 (29.0)	
	Yes	267 (71.0)	
**Previously diagnosed with STI^c^, n (%)**			
	No	273 (72.6)	
	Yes	103 (27.4)	
**Ever used pre-exposure prophylaxis, n (%)**			
	No	350 (93.1)	
	Yes	26 (6.9)	
**Ever used postexposure prophylaxis, n (%)**			
	No	349 (92.8)	
	Yes	27 (7.2)	
**Experienced childhood physical abuse, n (%)**			
	No	199 (52.9)	
	Yes	177 (47.1)	
**Experienced childhood sexual assault, n (%)**			
	No	235 (62.5)	
	Yes	141 (37.5)	
**Depressive symptoms, n (%)**			
	No	154 (41.0)	
	Yes	222 (59.0)	
**Ever injected drugs, n (%)**			
	No	359 (95.5)	
	Yes	17 (4.5)	
**Engaged in anal sex (past 6 months), n (%)**			
	No	13 (3.5)	
	Yes	363 (96.5)	
**Type of anal sex (past 6 months)^d^, n (%)**			
	Insertive	271 (72.1)	
	Receptive	285 (75.8)	
**HIV-serodiscordant relationship (past 6 months), n (%)**			
	No	340 (90.4)	
	Yes	36 (9.6)	
**Consistent condom use (past 6 months), n (%)**			
	No	304 (80.9)	
	Yes	72 (19.1)	
**Ever engaged in sexualized drug use, n (%)**			
	No	294 (78.2)	
	Yes	82 (21.8)	

^a^This category included college, university, or professional degrees.

^b^MYR: Malaysian Ringgit (1 MYR is approximately US $0.23).

^c^STI: sexually transmitted infections.

^d^The total exceeds 100% because the options were not mutually exclusive.

### Access to and Frequency of Use of Communication Technology

Almost all participants (368/376, 97.9%) owned or had access to a smartphone with internet access, and 13.3% (50/376) of participants had access to a basic cell phone without internet access (Table 2). More than two-thirds of participants (270/376, 71.8%) reported having access to a laptop; between one-quarter and one-fifth of participants had access to a personal computer (100/376, 26.6%), a tablet (85/376, 22.6%), and a landline telephone (81/376, 21.6%). The frequency of use of each device was largely consistent with the frequency of ownership and access, with smartphones representing the most frequently used technology (mean 4.9, SD 0.4), followed by laptops (mean 3.8, SD 1.2), personal computers (mean 2.5, SD 1.4), tablets (mean 2.3, SD 1.3), basic cell phones (mean 2.1, SD 1.3), and landline telephones (mean 1.8, SD 0.9).

Almost all participants (373/376, 99.2%) accessed the internet daily, largely through a smartphone (334/376, 88.8%), and spent on average 9.4 hours per week (SD 4.9) on the internet (Table 3). The most common activities that participants used the internet on their smartphones for were social networking (mean 4.5, SD 0.8) and sending or receiving emails (mean 4.0, SD 1.0). Participants also used their smartphones to access geosocial networking apps or websites (mean 3.6, SD 1.1), search for health-related information (mean 3.5, SD 0.9), and use health-related apps (mean 2.9, SD 1.1). The majority (345/376, 91.8%) of participants used geosocial networking apps, with Grindr, Blued, and Hornet being the most popular.

**Table 2:** Ownership or access to and frequency of use of communication technology.

Variable		Ownership or access (n=376), n (%)	Frequency of use^a^, mean (SD)
**Mobile phone**			
	With internet access (smartphone)	368 (97.9)	4.9 (0.4)
	Without internet access (basic phone)	50 (13.3)	2.1 (1.3)
Laptop		270 (71.8)	3.8 (1.2)
Personal computer		100 (26.6)	2.5 (1.4)
Tablet		85 (22.6)	2.3 (1.3)
Landline telephone		81 (21.5)	1.8 (0.9)

^a^This was assessed using a 5-point Likert scale (1, never; 2, rarely; 3, sometimes; 4, often; 5, all the time).

**Table 3:** Access to internet.

Variables			Respondents (n=376)
**Daily access to the internet**, **n (%)**			
	No	3 (0.8)	
	Yes	373 (99.2)	
**Primary device for accessing the internet, n (%)**			
	Smartphone	334 (88.8)	
	Laptop	21 (5.6)	
	Personal computer	6 (1.6)	
	Others	15 (4.0)	
Time spent on the internet (hours per week), mean (SD)			9.4 (4.9)
**Use of the internet on a smartphone for various activities^a^, mean (SD)**			
	Online social networking	4.5 (0.8)	
	Send or receive emails	4.0 (1.0)	
	Geosocial networking apps or websites	3.6 (1.1)	
	Search for health-related information	3.5 (0.9)	
	Use health-related apps	2.9 (1.1)	

^a^This item was assessed using a 5-point Likert scale (1, never; 2, rarely; 3, sometimes; 4, often; 5, all the time).

### Acceptability of mHealth

The majority of participants were interested in receiving HIV prevention information (345/376, 91.8%) on a monthly (147/376, 42.6% of those who expressed willingness) or weekly (131/376, 38.0%) basis, and in receiving medication reminders (336/376, 89.4%) mostly on a daily basis (191/376, 56.8%). Additionally, there was interest in using mHealth to screen and monitor sexual activity (306/376, 81.4%) on a weekly (135/376, 44.1%) or monthly (99/376, 32.4%) basis; screen and monitor illicit drug use (281/376, 74.7%) on a weekly (104/376, 37.0%) or monthly (100/376, 35.6%) basis; and monitor drug cravings (280/376, 74.5%) on a weekly (115/376, 41.1%) or monthly (79/376, 28.2%) basis. The preferred modality of mHealth strategies was via apps, regardless of the type of intervention (Table 4).

**Table 4:** Interest in and acceptance of mobile health (mHealth) among participants (N=376).

Interest in using mHealth to...					No, n (%)			Yes, n (%)
**Receive medication reminders**					40 (10.6)			336 (89.4)
	**Preferred frequency (n=374)**							
		Daily	13 (32.5)			191 (56.8)		
		Weekly	10 (25.0)			91 (27.1)		
		Monthly	8 (20.0)			44 (13.1)		
		Never	8 (20.0)			9 (2.7)		
	**Preferred mechanism (n=375)**							
		Phone calls	10 (25.0)			15 (4.5)		
		Text messages	13 (32.5)			93 (27.7)		
		App notification	12 (30.0)			206 (61.3)		
		Email	4 (10.0)			22 (6.5)		
**Monitor drug cravings**					96 (25.5)			280 (74.5)
	**Preferred frequency (n=375)**							
		Daily	10 (10.4)			48 (17.1)		
		Weekly	17 (17.7)			115 (41.1)		
		Monthly	15 (15.6)			79 (28.2)		
		Never	53 (55.2)			38 (13.6)		
	**Preferred mechanism (n=375)**							
		Phone calls	11 (11.5)			2 (0.7)		
		Text messages	23 (24.0)			65 (23.2)		
		App	45 (46.9)			183 (65.4)		
		Email	16 (16.7)			30 (10.7)		
**Screen and monitor illicit drug use**					95 (25.3)			281 (74.7)
	**Preferred frequency (n=375)**							
		Daily	10 (10.5)			50 (17.8)		
		Weekly	17 (17.9)			104 (37.0)		
		Monthly	17 (17.9)			100 (35.6)		
		Never	50 (52.6)			27 (9.6)		
	**Preferred mechanism (n=375)**							
		Phone calls	9 (9.5)			5 (1.8)		
		Text messages	22 (23.2)			55 (19.6)		
		App	45 (47.4)			189 (67.3)		
		Email	18 (18.9)			32 (11.4)		
**Screen and monitor sexual activity**					70 (18.6)			306 (81.4)
	**Preferred frequency (n=373)**							
		Daily	10 (14.3)			58 (19)		
		Weekly	15 (21.4)			135 (44.1)		
		Monthly	17 (24.3)			99 (32.4)		
		Never	26 (37.1)			13 (4.2)		
	**Preferred mechanism (n=375)**							
		Phone calls	8 (11.4)			5 (1.6)		
		Text messages	15 (21.4)			59 (19.3)		
		App	32 (45.7)			212 (69.3)		
		Email	14 (20.0)			30 (9.8)		
**Receive HIV prevention information**					31 (8.2)			345 (91.8)
	**Preferred frequency (n=374)**							
		Daily	6 (19.4)			53 (15.4)		
		Weekly	6 (19.4)			131 (38.0)		
		Monthly	10 (32.3)			147 (42.6)		
		Never	8 (25.8)			13 (3.8)		
	**Preferred mechanism (n=375)**							
		Phone calls	4 (12.9)			13 (3.8)		
		Text messages	5 (16.2)			70 (20.3)		
		App	12 (38.7)			186 (53.9)		
		Email	9 (29)			76 (22)		

### Correlates of mHealth acceptance

The mean score for mHealth acceptance was 4.1 (SD 1.5), with α=.875. In the multivariable model, being a university graduate (β=0.456, *P*=.003), being in a relationship with a partner (β=0.322, *P*=.04), lifetime engagement in sexualized drug use (β=0.489, *P*=.01), and recent engagement in receptive anal sex (β=0.498, *P*=.006) were associated with higher willingness to use mHealth strategies (Table 5).

**Table 5:** Univariate and multivariable linear regression correlates of mobile health acceptance among participants (n=376).

Variables			Univariate						Multivariable				
			Beta		SE		*P* value		Beta		SE		*P* value
Age (years)			–0.008		0.012		.52		—^a^		—		—
Ethnicity (Malaya)			0.145		0.160		.37		—		—		—
University graduate^b^			0.465		0.158		.003		0.456		0.154		.003
Relationship status (partner)			0.333		0.158		.04		0.322		0.154		.04
Monthly income			–0.001		0.001		.70		—		—		—
Ever had HIV test			–0.003		0.174		.99		—		—		—
Previously diagnosed with STI^c^			0.039		0.117		.82		—		—		—
Ever used pre-exposure prophylaxis			0.164		0.311		.60		—		—		—
Ever used postexposure prophylaxis			0.034		0.306		.91		—		—		—
Experienced childhood physical abuse			0.163		0.158		.30		—		—		—
Experienced childhood sexual assault			–0.017		0.163		.92		—		—		—
Depressive symptoms			0.253		0.160		.12		—		—		—
Ever injected drugs			0.678		0.379		.07		0.408		0.389		.29
Engaged in anal sex (past 6 months)			0.599		0.432		.17		—		—		—
**Type of anal sex (past 6 months)**													
	Insertive	–0.036		0.176		.84		—		—		—	
	Receptive	0.502		0.183		.006		0.498		0.179		.006	
HIV-serodiscordant relationship (past 6 months)			–0.314		0.268		.24		—		—		—
Consistent condom use (past 6 months)			0.130		0.201		.52		—		—		—
Ever engaged in sexualized drug use			0.537		0.189		.005		0.489		0.195		.01

^a^No data because the variable was not included in the model.

^b^This category included college, university, or professional degrees.

^c^STI: sexually transmitted infection.

## Discussion

### Principal Findings

The rapid growth and use of web-based communication technologies have led to innovations in public health programming and patient care [28], particularly as COVID-19–related restrictions affect health care delivery, with decreased access to in-person health care and prevention interventions, which has had negative consequences on health [43-47]. The utilization of innovative tools in virtual spaces (eHealth or mHealth) can help bridge gaps in service delivery that are needed to improve access to needed health and prevention services, particularly among underserved populations [26]. Until now, there has been a lack of empirical evidence on how communication technologies can improve access to and engagement in care or support for the use of mHealth strategies for HIV prevention needs among men who have sex with men in Malaysia. The findings from this study allow us to assess opportunities to implement mHealth strategies for HIV prevention efforts and inform the specific format and features of a mHealth platform tailored to the needs of men who have sex with men in Malaysia. We found near-universal access to communication technology and internet use and high levels of acceptability of mHealth, particularly smartphone apps, for HIV prevention efforts. Our findings provide preliminary evidence that supports the feasibility of mHealth deployment to deliver HIV prevention and related interventions among men who have sex with men in Malaysia.

We identified a number of interventions that can be incorporated into HIV-prevention mHealth, including tracking and monitoring the sexual and drug use behaviors and assessing symptoms associated with depression. Consistent with information in existing literature [48-50], a considerable proportion of men who have sex with men in our sample had high rates of condomless sex, sexualized drug use, and mental health comorbidities and sexually transmitted infections, highlighting the vulnerabilities in men who have sex with men in an evolving and dynamic HIV epidemic. In addition, it was concerning that a proportion of participants had never been tested for HIV (109/376, 29.0%). HIV testing is the first step in engaging individuals in HIV prevention and treatment cascades; frequent screenings are thus of the utmost importance for individuals most at risk. mHealth can play a uniquely important role in assessing risk and reminding and motivating men who have sex with men to be tested regularly [51-55]. As such, innovations in mHealth can accelerate engagement in HIV testing and facilitate the uptake of HIV prevention services, such as pre-exposure prophylaxis and HIV self-testing, particularly in areas where such services are underutilized or have limited availability [53]. Our findings underscore the urgent need for innovative strategies to reach and deliver HIV prevention services to this key population, particularly in a context where same-sex sexual behavior is deeply stigmatized.

Near-universal access to smartphones, combined with daily internet access among our sample, consistent with previous studies [56] with Malaysian men who have sex with men, support the feasibility of developing mHealth strategies for Malaysian men who have sex with men. Participants reported already using their smartphones to search for health-related information or apps, and most participants used mobile technologies (ie, smartphones) far more frequently than they used other technologies (eg, computers, landline telephone). This likely reflects the digital revolution that is especially explosive in Asia and the fast-paced growth and use of mobile technologies in the community [57-61]. The rapid advances in mobile technologies and the development of apps open new opportunities for integrating mobile health into existing HIV prevention service delivery in the region. Future research, however, is needed to gather additional information on Malaysian men who have sex with men’s interests in health-related content using smartphones, access points (eg, websites, chatbots, apps), which types of information, and where gaps in existing web-based resources exist.

Our findings indicate that there is considerable interest in specific mHealth strategies, such as receiving information related to HIV (345/376, 91.8%), receiving medication reminders (336/376, 89.4%), screening and monitoring sex activity (306/376, 81.4%) and illicit drug use (281/376, 74.7%), and monitoring drug cravings (280/376, 74.5%). In the absence of public dialogue about these issues in Malaysia, it is not surprising that Malaysian men who have sex with men, similar to men who have sex with men in other settings [62-67], and specifically, for HIV prevention, in China [68], Vietnam [69], and Indonesia [66], expressed interest. In our sample, men expressed interest in the use of mHealth apps to receive daily reminders to take medications; however, weekly and monthly reminders were preferred for other activities, with smartphone apps being the preferred platform. An mHealth-based app may serve as an additional tool that can help support men who have sex with men with HIV prevention or care needs between clinical visits, guide them to needed services, enhance clinical care and support through screening and recommendations, and provide different modes of accessing information, services, and prevention commodities. Men who have sex with men at higher risk for HIV due to sexual practices, such as recent engagement in receptive anal sex and lifetime engagement in sexualized drug use, were more willing to use mHealth-based strategies, which suggests that mHealth approaches are particularly promising to address the needs of the subset of men who have sex with men who would most benefit.

While this study focused on HIV prevention behavior, knowledge, and access to services among HIV-negative Malaysian men who have sex with men, mHealth-based health intervention strategies can potentially also play an important role among HIV-positive men who have sex with men, for instance, by encouraging adherence to antiretroviral therapy and increasing retention in the HIV-care cascade [70]. High rates of mHealth acceptability have been reported in HIV-positive men who have sex with men and transgender women in Malaysia, for receiving HIV-related and sexual health information, assessing sexual and health behaviors, and receiving reminders to take HIV medications [71].

### Limitations

Despite the many new findings, this study is not without limitations. First, we used self-reported measures for HIV and sexually transmitted infection diagnoses, sexual behavior, and drug use, which may have introduced some social desirability bias and underreporting of stigmatized behaviors. We reduced the potential for social desirability bias by designing the questionnaire to be self-administered and anonymous and by allowing participants to complete the questionnaire alone (without the presence of research staff). Second, though participants were recruited using respondent-driven sampling, some data suggest that a representative sample of all men who have sex with men in Malaysia may not have been achieved. For example, over half of our participants were university graduates, compared with only one-sixth among the general adult population in Malaysia [72]. Men who have sex with men in Malaysia’s capital, however, are typically more educated, which perhaps makes the sample more representative of urban men who have sex with men in Malaysia; our findings on participants’ educational status are consistent with previous studies conducted among men who have sex with men in the region [49,71]. The educational level of our sample may explain the high level of interest in mHealth and the acceptability of mHealth interventions, as a higher level of education likely facilitates greater technology literacy. This is supported by the multivariable analysis of associations between mHealth acceptance and participant characteristics, which showed that university graduates were more willing to use mHealth strategies. Finally, although men in this study showed a strong willingness to use mHealth for various needs, it should be noted that willingness or interest may not fully reflect actual use; therefore, studies of mHealth interventions in practice are needed to assess use.

To the best of our knowledge, this is the first study to assess the acceptability of mHealth to address HIV prevention needs among men who have sex with men in Malaysia. Our findings show that mHealth use, particularly app-based platforms, appears to be highly acceptable to this population. This finding is particularly meaningful in Malaysia, where there are limited physical venues that are culturally acceptable for men who have sex with men to seek health care since same-sex sexual behavior is illegal, and men who have sex with men are highly stigmatized and are frequent targets of discrimination [9].

### Future Implications

Our findings support the development of mHealth-based strategies, especially smartphone apps, to jumpstart the HIV prevention cascade by promoting HIV testing and, depending on the results, linking individuals to the appropriate prevention or treatment services. mHealth strategies, such as culturally tailored apps, are uniquely positioned to deliver multicomponent interventions, and thus, can bridge systematic gaps needed to address syndemic and complex interrelated health needs and more effective utilization of health services in this underserved population [53,55,73-76]

In recent years, several apps for HIV prevention and treatment efforts have been evaluated in pilot studies or randomized trials that incorporate components such as HIV testing, condom use, pre-exposure prophylaxis, treatment as prevention, and other support services (eg, mental health, drug use) [53,55,73-76]. Some of these apps offer the opportunity to assess, with ecological momentary assessment, or intervene, via ecological momentary interventions, individuals in their natural environment, thereby enabling a better understanding of the factors triggering problems and addressing the problems when and where they arise [77-79].

Most, if not all, of the available apps, however, are primarily developed for use in high-income countries [53,55,73-76]. Additional research to assess the design, functionality, and content preferences of Malaysian men who have sex with men is now needed to facilitate the design of a customized mHealth app in the Malaysian context (specifically, addressing the multiethnic population of Malaysia, as well as men who have sex with men in nonurban settings, will be important). Further research is also needed to understand the perspective of those tasked with providing care and support services via the mHealth platforms (eg, physicians, pharmacists, counselors, outreach workers). Such research will inform and facilitate the integration of mHealth platforms within existing health care services.

## Abbreviations

mHealth: mobile health
MYR: Malaysian Ringgit
